# Piecing Together How Peroxiredoxins Maintain Genomic Stability

**DOI:** 10.3390/antiox7120177

**Published:** 2018-11-28

**Authors:** James D. West, Trevor J. Roston, Joseph B. David, Kristin M. Allan, Matthew A. Loberg

**Affiliations:** Biochemistry & Molecular Biology Program, Departments of Biology and Chemistry, The College of Wooster, Wooster, OH 44691, USA; roston1@live.marshall.edu (T.J.R.); jdavid15@wooster.edu (J.B.D.); kallan16@wooster.edu (K.M.A.); mloberg16@wooster.edu (M.A.L.)

**Keywords:** peroxiredoxin, oxidative stress, thioredoxin, thiol peroxidase, mutator, genomic instability, sulfiredoxin, redox switch, ribonucleotide reductase

## Abstract

Peroxiredoxins, a highly conserved family of thiol oxidoreductases, play a key role in oxidant detoxification by partnering with the thioredoxin system to protect against oxidative stress. In addition to their peroxidase activity, certain types of peroxiredoxins possess other biochemical activities, including assistance in preventing protein aggregation upon exposure to high levels of oxidants (molecular chaperone activity), and the transduction of redox signals to downstream proteins (redox switch activity). Mice lacking the peroxiredoxin Prdx1 exhibit an increased incidence of tumor formation, whereas baker’s yeast (*Saccharomyces cerevisiae*) lacking the orthologous peroxiredoxin Tsa1 exhibit a mutator phenotype. Collectively, these findings suggest a potential link between peroxiredoxins, control of genomic stability, and cancer etiology. Here, we examine the potential mechanisms through which Tsa1 lowers mutation rates, taking into account its diverse biochemical roles in oxidant defense, protein homeostasis, and redox signaling as well as its interplay with thioredoxin and thioredoxin substrates, including ribonucleotide reductase. More work is needed to clarify the nuanced mechanism(s) through which this highly conserved peroxidase influences genome stability, and to determine if this mechanism is similar across a range of species.

## 1. Factors Influencing Genomic Stability

Numerous safeguards help minimize the accumulation of mutations in actively dividing cells. DNA polymerases, DNA repair proteins, cell cycle regulators, signaling proteins that sense and respond to DNA damage, and detoxification enzymes that minimize environmental DNA damage all contribute to the faithful transmission of genetic material from mother to daughter cell during each cell division [1]. Despite this, the fidelity of DNA replication and chromosomal division can be compromised in certain situations. For instance, cancers are commonly associated with genomic instability—the inability of a progenitor cell to pass down a genome containing minimal alterations to its daughter cells [2,3,4,5]. This increase in genomic instability is likely due to the age-associated accumulation of gain-of-function mutations in oncogenes that accelerate cell division, and inactivating mutations in key tumor suppressors that confer genomic stability and/or slow cell cycle progression [4,6]. 

While a number of individual genetic and pharmacological studies have pinpointed particular proteins and pathways that contribute to genome stability, surprisingly few genome-wide screens have been performed to identify factors that influence mutation rates in different model systems. The majority of this work has been completed in the baker’s yeast *Saccharomyces cerevisiae*. In this system, the availability of genetic tools, and a suite of phenotypic assays for measuring mutation rates and gross chromosomal perturbations makes identifying factors that contribute to genome stability possible on a whole genome scale [7,8]. Indeed, both protein over-expression screens and screening of yeast deletion mutants have led to the identification of numerous factors that influence mutation rates and gross chromosomal rearrangements in cells [9,10,11,12,13]. Due to the limitation that genome-wide screens which use deletion strains are often restricted to nonessential genes, we only have a partial list of the factors that participate in the faithful copying and transmission of genetic material to daughter cells. Even with this limited data set, several classes of nonessential genome stability regulators have emerged, including DNA repair proteins, DNA replication factors, and proteins that protect against damage by reactive oxygen species [9,10]. 

Of the oxidant defense genes contributing to genomic stability, the peroxiredoxin Tsa1, an abundant thiol-dependent oxidoreductase, is the strongest suppressor of mutations [9]. Cells lacking Tsa1 exhibit approximately a 5–10-fold increase in mutation rates depending on the assay [9]. Specifically, deletion of *TSA1* leads to an increase in a variety of genome perturbations, including single and double nucleotide substitutions, insertion/deletion mutations, and gross chromosomal rearrangements [9,10,14]. In contrast, other peroxidases (e.g., other peroxiredoxins, glutathione peroxidases, and catalases) contribute minimally to suppressing mutations when individually deleted [9], and only a modest enhancement in mutation rates, if any, is observed when other peroxiredoxins are deleted in combination with *TSA1* [14,15]. The molecular mechanism employed by Tsa1 in mutation suppression (in addition to other cellular phenotypes) has yet to be clearly defined, given that the protein exists in multiple structural states and has at least three different redox-dependent biochemical activities [16]. To better appreciate the potential means through which Tsa1 decreases mutation rates, a review of the protein’s structural transformations and biochemical activities is warranted. 

## 2. Multistep Catalytic Cycle of Typical 2-Cys Peroxiredoxins

**Figure 1:**
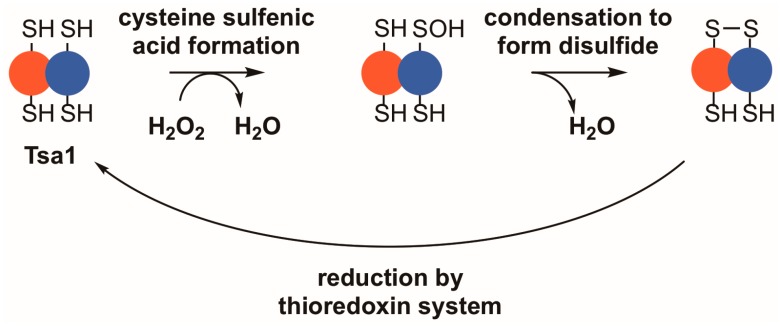
Tsa1 and other typical 2-Cys peroxiredoxins detoxify peroxides through a thiol-dependent, disulfide-based mechanism. Upon binding a peroxide, Tsa1 is oxidized on its peroxidatic cysteine to form a cysteine sulfenic acid (–SOH). Subsequently, the sulfenic acid condenses with the thiol in the resolving cysteine to form a disulfide bond. Thioredoxins reduce the resulting disulfide bond to complete the catalytic cycle.

While all peroxiredoxins employ a thiol-based mechanism to detoxify peroxides, Tsa1 falls into a highly conserved class of peroxiredoxins—the typical 2-Cys peroxiredoxins—which possess distinct biochemical and structural properties [17,18,19]. In their reduced state, members of this class of peroxiredoxins most often form toroid-shaped decamers, assembled from five sets of dimers [20,21,22,23,24,25]. Two active sites reside between each subunit pair, on either side of the dimer interface, where they can reduce a variety of different substrates, including inorganic peroxides, aliphatic hydroperoxides, or hydroperoxides formed by oxidation of amino acids, peptides, or proteins [26,27,28]. Within each active site, a peroxidatic cysteine with a depressed thiol pK_a_ purportedly attacks a substrate and directly reduces the peroxide to water or an alcohol [29,30]. The reaction with peroxide occurs at rates near the limit of diffusion, suggesting that peroxiredoxins in this class are key oxidant defense enzymes [29,31,32]. Upon oxidation of the peroxidatic cysteine thiol, a sulfenic acid is formed; subsequently, the active site region undergoes a local unfolding event that allows a thiol group on the resolving cysteine from the adjacent subunit to condense with the sulfenic acid, forming an inter-subunit disulfide bond (Figure 1) [30,33]. The decamer weakens following disulfide formation, leading to disassembly into dimers in some instances [22,25,34,35]. To be restored to its catalytically active form, the protein is commonly reduced by thioredoxin, a general protein disulfide reductase, which carries out a disulfide exchange with the peroxiredoxin or other disulfide-containing proteins [36,37,38]. Subsequently, the oxidized form of thioredoxin is reduced by thioredoxin reductase using reduced nicotinamide adenine dinucleotide phosphate (NADPH) as the principal reductant for the completion of the peroxiredoxin catalytic cycle [39,40]. 

In terms of the reductive half-reactions, several studies have indicated that the cytosolic typical 2-Cys peroxiredoxins, like Tsa1, are major substrates for the cytosolic thioredoxin machinery. In baker’s yeast, for example, Tsa1 is the most abundant peroxiredoxin [41]. It makes up approximately 1% of the cytosolic protein content, and is estimated to be present at 10–50 µM (based on quantitative proteomic measurements) [42,43,44]. Thus, the sheer abundance of Tsa1 may divert thioredoxin away from other disulfide-containing proteins under homeostatic conditions and during oxidative stress. In support of this, we have found Tsa1 readily undergoes cross-linking to thioredoxin in yeast cells treated with the bifunctional electrophile divinyl sulfone, unlike many other cytosolic thioredoxin substrates [45,46]. These in vivo cross-linking results indicate that Tsa1 and thioredoxin interact with one another frequently and/or tightly in cells. Likewise, the cytosolic 2-Cys peroxiredoxin Tpx1 is thought to be the principal substrate of thioredoxin in *Schizosaccharomyces pombe* [47,48]. Moreover, homologous peroxiredoxins Prdx1 and Prdx2 are expressed at high concentrations in mammalian cells. For example, Prdx1 and Prdx2 have an estimated expression of 120 µM and 50 µM in Jurkat T cell lymphoma cells, respectively. Prx2 concentrations in erythrocytes reach approximately 240 µM, making it the third most abundant protein in this cell type [49,50]. Collectively, these findings suggest a conserved, intricate partnership between the typically abundant 2-Cys peroxiredoxins and corresponding cytosolic thioredoxins during both homeostasis and oxidative stress. 

## 3. Reversible Hyperoxidation and Chaperone Activity of Peroxiredoxins

**Figure 2:**
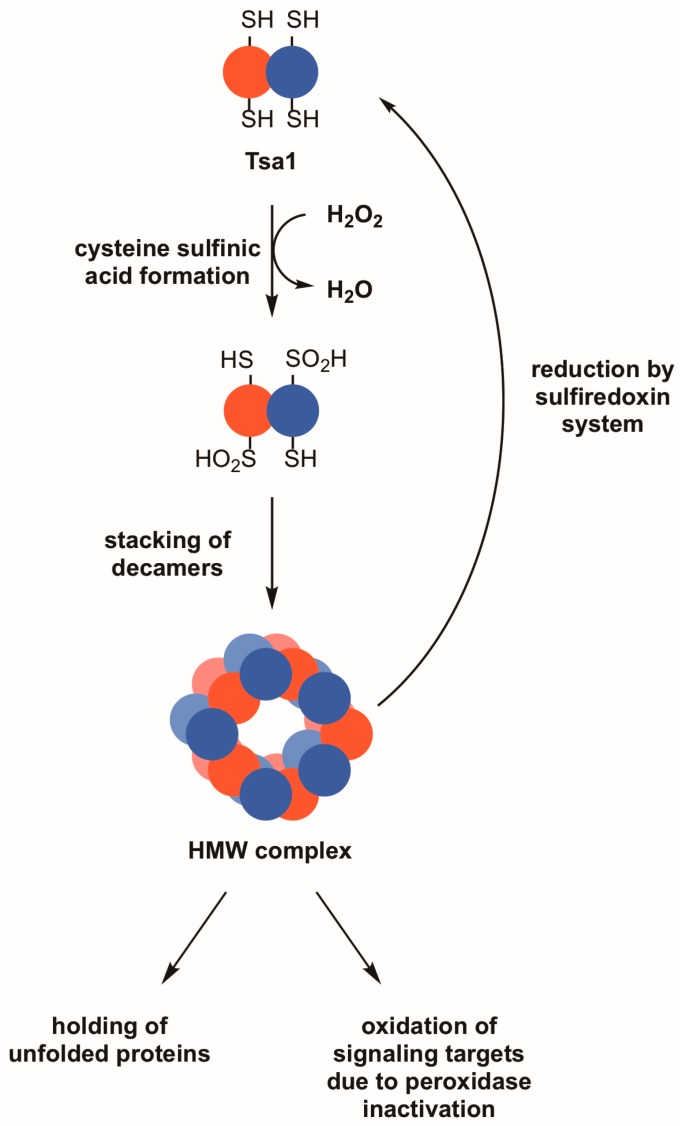
Reversible hyperoxidation of Tsa1 and other 2-Cys peroxiredoxins leads to the acquisition of molecular chaperone activity and may result in the oxidation of other proteins. During periods of pronounced oxidative stress, the thiol group in the peroxidatic cysteine of Tsa1 and similar peroxiredoxins can form cysteine sulfinic acid (–SO_2_H). This hyperoxidation event can cause Tsa1 decamers to oligomerize into high molecular weight (HMW) complexes, which are thought to possess molecular chaperone activity as holdases. An indirect effect of Tsa1 inactivation is that other proteins that are normally less sensitive to peroxide become oxidized. Hyperoxidized Tsa1 can be restored to its functional form by the combined actions of the sulfiredoxin and thioredoxin systems.

In certain instances, the peroxidatic cysteine found in eukaryotic 2-Cys peroxiredoxins of the typical class does not resolve quickly to the disulfide following sulfenic acid formation [51]. Such a long-lived sulfenic acid intermediate can undergo further oxidation to form a cysteine sulfinic acid and, in extreme cases, a cysteine sulfonic acid. The sulfinic acid oxidation state was considered irreversible for many years; however, a sulfinic acid reductase that recognizes and reduces hyperoxidized peroxiredoxins—sulfiredoxin—exists in diverse eukaryotic species [52]. Sulfiredoxins from different species catalyze the ATP-dependent reduction of cysteine sulfinic acids in peroxiredoxins, undergoing oxidation on their catalytic cysteine residues (Figure 2) [53,54]. The enzyme must subsequently be reduced either by thioredoxin or glutathione, depending on the organism [54,55].

Hyperoxidation of peroxiredoxins is postulated to have both direct and indirect functional consequences. The hyperoxidized form of peroxiredoxins either remains in a decameric state or oligomerizes further to a high molecular weight (HMW) state (e.g., a 20-mer consisting of two stacked decamer rings) [56,57]. Regardless of the subunit composition, the large complex transitions from an active peroxidase involved in peroxide detoxification to a molecular chaperone with holdase activity (Figure 2) [56,57,58]. Tsa1 associates with several ribosomal proteins following proteotoxic stress, suggesting that it prevents these subunits from undergoing stress-induced aggregation and/or decreases unwanted oligomerization of nascent peptides that emerge from the ribosome under these conditions [59]. An alternative view posited for the hyperoxidation of peroxiredoxins is that, with their inactivation, peroxides are more available to oxidize other intracellular target proteins as a means of redox signaling (Figure 2) [51]. This has been suggested because typical 2-Cys peroxiredoxins from eukaryotes are more susceptible to hyperoxidation, suggesting that, when they are in their inactive, sulfinic acid forms, more peroxides are available to oxidize other protein targets in a cell [51]. There is only limited biological evidence to support the latter ‘floodgate’ model wherein peroxiredoxin hyperoxidation and inactivation lead to oxidative regulation of other proteins [60], but the direct and indirect effects associated with the hyperoxidation and inactivation of peroxiredoxins are worth considering when seeking an explanation for their role in maintaining genome stability. 

## 4. Direct Redox Signaling by Typical 2-Cys Peroxiredoxins

In addition to their direct role in oxidant defense and their molecular chaperone activity, peroxiredoxins from a variety of species have been implicated in directly and indirectly transducing redox signals [61,62,63]. Peroxiredoxins can form mixed disulfides with various signaling partners following sulfenic acid formation or undergo a disulfide exchange with a redox partner besides thioredoxin [64,65]. In addition, peroxiredoxins may indirectly promote oxidation of other proteins by keeping thioredoxin in an oxidized (therefore, inactive) state [66,67,68,69]. Peroxiredoxin-mediated redox signaling occurs in fission yeast *Schizosaccharomyces pombe*, where the antioxidant response transcription factor Pap1 is directly oxidized by the peroxiredoxin Tpx1 [70,71]. In mammalian cells, regulation of the JAK-STAT pathway is mediated by direct transmission of redox signals from Prdx2 to STAT3 [72]. In baker’s yeast, however, redox signaling by typical 2-Cys peroxiredoxins is less clearly defined. In specific strains of *S. cerevisiae*, which lack an intact glutathione peroxidase 3 (Gpx3) signaling pathway, Tsa1 can substitute for Gpx3 as an activator of the oxidant-sensing transcription factor Yap1 [41,73]. The activities of other stress-responsive transcription factors (e.g., Msn2 and Skn7) are also influenced by Tsa1 in certain instances [74,75], although the mechanisms through which their activation occurs need further investigation. Perhaps there are one or more protein signaling targets of Tsa1 and related peroxiredoxins that undergo redox-dependent activation to influence genome stability, thereby lowering mutations. 

## 5. Relating the Biochemical Activities of Tsa1 to Genomic Stability

**Figure 3:**
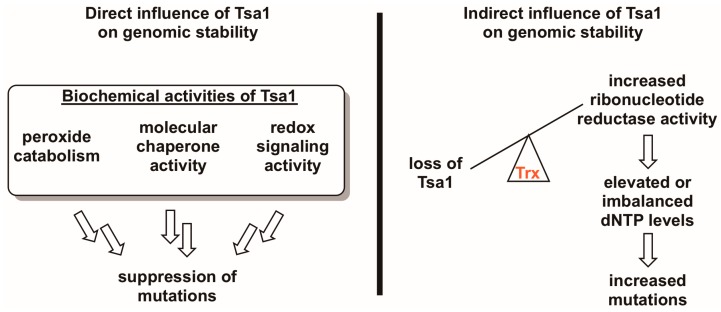
Tsa1 may influence mutation rates in a direct and/or an indirect way. Given the three biochemical activities of Tsa1 and related peroxiredoxins (i.e., peroxide detoxification, molecular chaperone activity, and redox switch activity), it is possible that any of these directly contribute to suppressing mutations. However, there is evidence suggesting that Tsa1 may influence genomic stability by titrating thioredoxin away from other substrates involved in DNA synthesis or repair, most notably ribonucleotide reductase. In the case of the Tsa1-ribonucleotide reductase competition hypothesis, Tsa1-mediated thioredoxin (Trx) sequestration may be a way of regulating ribonucleotide reductase activity, thereby maintaining appropriate dNTP levels and lowering mutation rates.

Because Tsa1 adopts a variety of redox-dependent structural states and possesses three biochemical activities, determining the mechanism through which it lowers the incidence of mutations has been a challenge. Here, we review results from a number of laboratories on Tsa1-mediated mutation suppression in relation to what we understand about its biochemical properties, positing potential direct and indirect mechanisms through which it lowers the incidence of mutations (Figure 3). In addition, we provide experimental insights from our own studies on this topic that are aimed at clarifying this complex phenotype. 

### 5.1. Monitoring Whether Tsa1 Influences Mutation Rates Based on Its Peroxidase Activity

Initially, it was inferred that, because Tsa1 is a peroxidase, it may lower the levels of oxidative DNA damage, thereby decreasing the incidence of mutations. Earlier studies provided a basis for such a hypothesis, since elevated expression of a related peroxiredoxin (AhpC) was associated with decreased oxidative DNA damage and mutagenesis in *Salmonella typhimurium* [76]. Moreover, embryonic fibroblasts isolated from Prdx1-deficient mice exhibit elevated levels of the oxidized DNA base 8-oxo-dG when compared with wild-type controls [77]. In support of the idea that Tsa1 decreases oxidative DNA damage to suppress mutations, the mutation spectrum that is observed with *TSA1* deletion is similar to that observed for the mutation spectrum that results from oxidant exposure [78]. In addition, Tsa1 was one of several oxidant defense factors, any of which could decrease the levels of mutagenic oxidized DNA bases, which yielded a mutator phenotype when deleted from yeast. The other genes that resulted in a mutator phenotype upon deletion included Yap1, Gpx3, the superoxide dismutase Sod1, the superoxide dismutase copper chaperone Lys7, and the 8-oxoguanine glycosylase Ogg1 [9]. Of these, combined deletion of *OGG1* and *TSA1* leads to a strong enhancement of mutation rates in some assays of genomic instability, implying that these enzymes may work together to suppress mutations [79]. Similarly, combined deletion of *TSA1* with other genes encoding proteins involved in repairing oxidative DNA damage (via base excision or nucleotide excision repair) enhances chromosomal instability [80]. Moreover, loss of both human Prdx1 and the 8-oxodeoxyguanosine triphosphatase MTH1 leads to increased levels of oxidized guanine in DNA, implying that human peroxiredoxins and enzymes that decrease oxidative DNA damage work collaboratively to lower mutation load [81]. 

**Figure 4:**
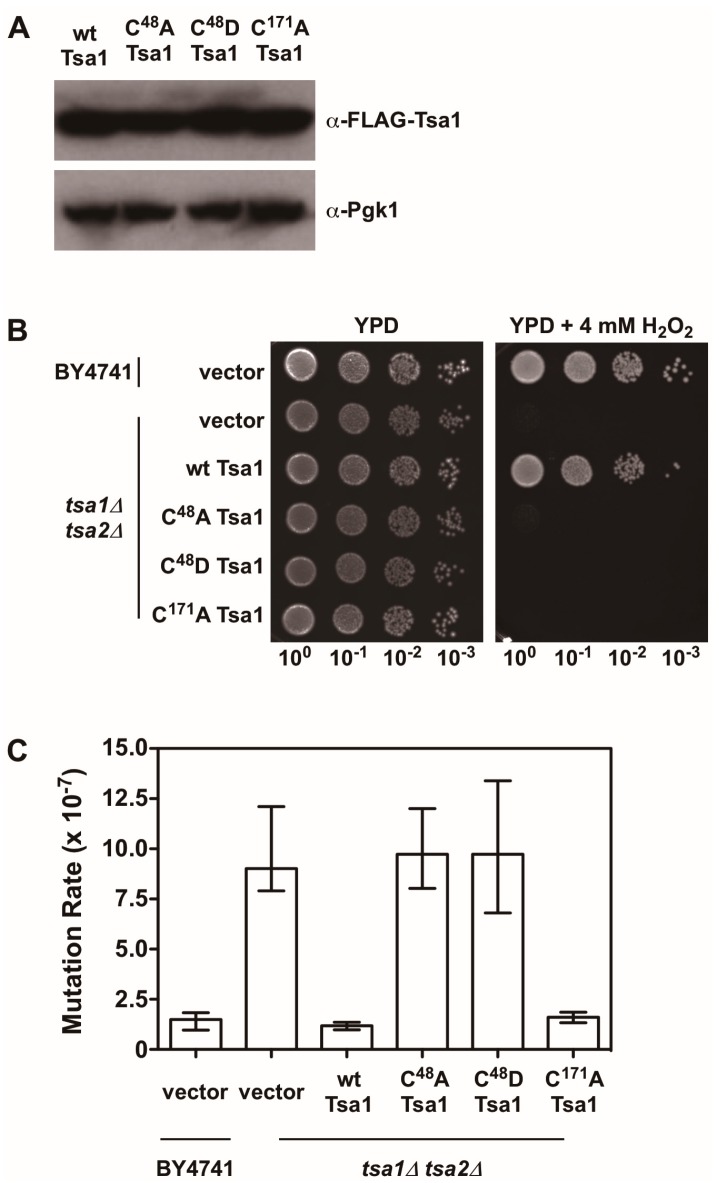
The peroxidatic cysteine in Tsa1 is required to protect against H_2_O_2_ and lower mutation rates. (**A**) Western blot analysis of FLAG-tagged Tsa1 variants. Pgk1 levels were monitored as a loading control. (**B**) Stationary phase cultures of wild-type (BY4741) cells transformed with vector, *tsa1*Δ *tsa2*Δ cells transformed with vector, or *tsa1*Δ *tsa2*Δ cells expressing FLAG-tagged Tsa1 variants were diluted serially, plated on non-selective growth medium (YPD) or YPD containing 4 mM H_2_O_2_, and grown for 48 h at 30 °C. Results are representative of three independent experiments. (**C**) Mutation rates in cells expressing Tsa1 variants were determined by monitoring fluctuation of the *CAN1* gene, as assessed by counting canavanine-resistant colonies for nine independent isolates of each strain in duplicate. The graph depicts the median mutation rate ± the 95% confidence limit for each strain. Detailed methods are available in Supplementary Materials.

**Figure 5:**
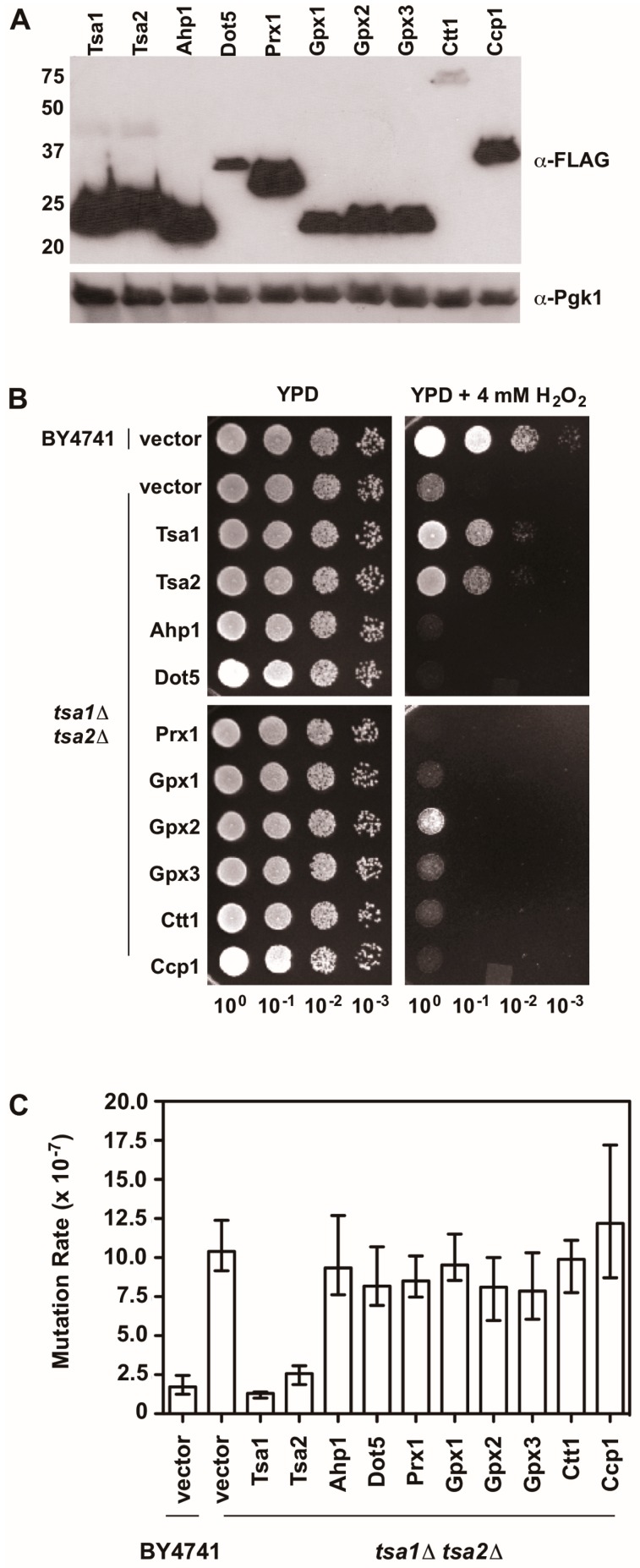
An overexpression screen of yeast peroxidases reveals that only Tsa1 and Tsa2 protect against H_2_O_2_ and suppress mutations in *tsa1*Δ *tsa2*Δ yeast. (**A**) Western blot analysis of FLAG-tagged peroxidases. Pgk1 levels were monitored as a loading control. (**B**) Stationary phase cultures of wild-type (BY4741) cells transformed with vector, *tsa1*Δ *tsa2*Δ cells transformed with vector, or *tsa1*Δ *tsa2*Δ cells expressing FLAG-tagged peroxidases were diluted serially, plated on non-selective growth medium (YPD) or YPD containing 4 mM H_2_O_2_, and grown for 48 h at 30 °C. Results are representative of three independent experiments. (**C**) Mutation rates in cells expressing various peroxidases were determined by monitoring fluctuation of the *CAN1* gene, as assessed by counting canavanine-resistant colonies for nine independent isolates of each strain in duplicate. The graph depicts the median mutation rate ± the 95% confidence limit for each strain. Detailed methods are available in Supplementary Materials.

There is also evidence indicating that Tsa1 suppresses mutations using a mechanism that is independent of oxidant-mediated DNA damage and mutagenesis. Along these lines, none of the oxidant defense factors identified in earlier screens are as strong being mutation suppressors as Tsa1 [9]. Moreover, expression of a catalytically dead form of Tsa1, wherein the resolving cysteine was substituted with serine or alanine, still suppressed mutation rates similar to wild-type Tsa1, despite being less capable of protecting against peroxide toxicity (Figure 4) [15,82]. In contrast, substitution of the cysteine that reacts with peroxide (C^48^) to serine or alanine did not rescue the mutator phenotype observed in yeast lacking *TSA1* and *TSA2* (*tsa1*Δ *tsa2*Δ; Figure 4) [15]. In addition, our laboratory found that over-expression of Tsa1 only, or the closely related isoform Tsa2, but not the majority of other peroxidases expressed in baker’s yeast, was capable of rescuing the mutator phenotype in the *tsa1*Δ *tsa2*Δ yeast strain (Figure 5). Presumably, one or more of the other peroxidases could lower the incidence of oxidative DNA damage that might be elevated in this genetic background. Of particular note, we overexpressed the cytochrome c peroxidase Ccp1, an enzyme that is amplified via aneuploidy to counteract the combined deletion of all eight thiol-based peroxidases in yeast [83], and observed that it had little effect on oxidant defense or mutation rates in yeast lacking both Tsa1 and Tsa2. While only Tsa1 and Tsa2 were capable of suppressing the mutator phenotype associated with *TSA1* loss, human Prdx1 was able to complement the mutator phenotype observed in this strain [84]. Taken together, these previously published and new results indicate that Tsa1 has a unique role among thiol-based peroxidases in promoting genome maintenance in yeast that does not require its full function as a peroxidase, and this role was preserved in related cytosolic, typical 2-Cys peroxiredoxins from other species. Therefore, it is important to consider other aspects of Tsa1 function that may influence this phenotype. 

### 5.2. Assessing the Role of Tsa1’s Chaperone Activity in Genomic Stability 

As noted earlier, upon hyperoxidation of the peroxidatic cysteine to a sulfinic or sulfonic acid, Tsa1 and related peroxiredoxins form HMW complexes that acquire holdase activity and prevent misfolded proteins from aggregating together [56,58]. Sulfiredoxin influences the persistence of these HMW complexes of Tsa1 following exposure to oxidants, implying that it suppresses Tsa1’s molecular chaperone activity [56]. Overexpression or deletion of *SRX1* in baker’s yeast does not alter mutation rates in general, suggesting that Tsa1 hyperoxidation and the corresponding molecular chaperone function do not contribute significantly to genome stability [85]. In addition, substituting the peroxidatic cysteine with aspartate is proposed to mimic the hyperoxidized state and leads to the acquisition of molecular chaperone activity in a peroxiredoxin from *Arabidopsis thaliana* [86]. We introduced a similar substitution into Tsa1 (C^48^D) and found that this form of Tsa1, like the C^48^A mutant, did not rescue the mutator phenotype or contribute to oxidant defense in *tsa1*Δ *tsa2*Δ yeast (Figure 4). It remains to be determined whether this Tsa1 variant possesses chaperone activity. Collectively, these different lines of evidence, while limited, suggest that the molecular chaperone function of Tsa1 does not contribute in an appreciable way to its role in genome maintenance. 

### 5.3. Determining Whether Tsa1 Acts as a Redox Switch to Influence Genome Stability

Tsa1 protects against oxidants due to its catalytic partnership with the thioredoxin system, but it may oxidize other proteins involved in signaling in certain cases. Although the signaling targets of Tsa1 and orthologous peroxiredoxins in other organisms are poorly understood, this ‘redox switch’ activity may be accomplished in one of two ways: (a) through direct condensation of the cysteine sulfenic acid with a signaling target to oxidize that protein, or (b) through a disulfide exchange with a signaling target through a similar mechanism as that carried out by thioredoxin [64]. If the first of these two options were to occur to activate a factor that promotes genomic stability, such a mechanism may help explain why Tsa1 without a resolving cysteine can still suppress mutations. To date, there is limited evidence to support such a model. We do note that the activity of several stress-responsive transcription factors from yeast—notably Skn7 and Yap1—exhibit mutator phenotypes and their activities are influenced by Tsa1 expression in some instances [73,74]. Likewise, the activity of transcription factor Msn2 is influenced by Tsa1 in a manner that requires both active site cysteines [75]. To our knowledge, though, no one has assessed whether a yeast strain lacking both Msn2 and Msn4 exhibits a mutator phenotype. In all cases, a direct link between Tsa1, these stress-responsive transcription factors, and their combined influence on genomic stability has not been established; therefore, additional work is needed to determine whether there is a redox switch activity that influences the function of these transcription factors or other proteins, thereby contributing to mutation suppression. 

## 6. Titrating Thioredoxin Away from Ribonucleotide Reductase as an Indirect Mechanism of Mutation Suppression by Peroxiredoxins

An alternative model for how Tsa1 lowers mutation rates, irrespective of whether it possesses full peroxidase activity, involves its relationship with the thioredoxin machinery. This model assumes that Tsa1 lacking a resolving cysteine still retains a similar affinity for thioredoxin as the wild-type protein, despite not being able to form an inter-subunit disulfide bond that is most commonly associated with thioredoxin-mediated reduction. As noted earlier, Tsa1 represents one of the most abundant thioredoxin substrates in the cytosol [61], and our group and others have proposed that peroxiredoxins in its class represent one of the major interaction partners of thioredoxins [45,47]. However, the steady-state expression levels of several other thioredoxin substrates are also on a similar scale, including those that are closely linked with genomic stability (e.g., the individual ribonucleotide reductase subunits Rnr1 and Rnr4) [43,44]. These results from quantitative global proteome profiling experiments suggest that Tsa1 and ribonucleotide reductase activities may be in a delicate balance with one another, a balance that depends on the amount of available thioredoxin as an electron donor [87]. 

Under homeostatic conditions, Tsa1 may limit overall ribonucleotide reductase activity, an enzyme complex that catalyzes the rate-limiting step in deoxyribonucleotide (dNTP) synthesis, in one of two ways. First, a fully active form of Tsa1 may lower the overall levels of DNA damage, as noted earlier. DNA damage of various types has been associated with imbalanced dNTP production in diverse species, perhaps due to the induced expression of ribonucleotide reductase subunits [88,89]. Tsa1, through its ability to decrease oxidative DNA damage and/or signal damage through a redox switch to downstream proteins, would prevent DNA damage response activation that in turn leads to enhanced ribonucleotide reductase activity. Second (and arguably more consistent with the available data), Tsa1 may act by titrating thioredoxin away from ribonucleotide reductase, due to its abundance (as suggested in fission yeast) [48]. When Tsa1 is no longer expressed, inappropriately regulated ribonucleotide reductase activity may lead to elevated dNTP levels or imbalanced ratios between individual dNTPs, both of which enhance the infidelity during replication and mutation rates [90]. Although this mechanism has not been studied in the context of Tsa1 deletion, dysregulation of ribonucleotide reductase activity using variants that are incapable of feedback inhibition by high dNTP levels enhances mutation rates [91,92]. Thus, this intricate relationship between Tsa1, ribonucleotide reductase, and thioredoxin provides another way of accounting for the mutator phenotype observed in yeast lacking Tsa1. 

In support of a genetic interaction between ribonucleotide reductase and Tsa1 in maintaining genome stability, combined deletion of *TSA1* and genes encoding the ribonucleotide reductase inhibitors Sml1 or Crt1 leads to enhanced mutation rates when compared with the wild-type strain or strains lacking these genes individually [93]. In contrast, deletion of both *TSA1* and *DUN1*, a positive regulator of ribonucleotide reductase activity, decreases mutation rates in some assays (e.g., canavanine fluctuation) but enhances the incidence of gross chromosomal rearrangements, suggesting a genetic interaction between Tsa1 and ribonucleotide reductase in controlling mutation rates [79,93]. In addition, dNTP levels are elevated and imbalanced in yeast lacking Tsa1, suggesting that ribonucleotide reductase activity is enhanced when Tsa1 is absent [93,94]. Moreover, loss of the gene encoding thioredoxin reductase *TRR1* decreases the enhanced mutation rates associated with *TSA1* deletion [95]. Whether the loss of Tsa1 increases the availability of thioredoxin for other tasks that destabilize the genome, or if the effect is instead related to the enhanced DNA damage response signaling in Tsa1-deficient yeast is unclear. Therefore, additional experimental evidence, both genetic and biochemical, is needed to determine the nature of the genetic interaction between Tsa1, thioredoxin, and ribonucleotide reductase on a molecular level. 

## 7. Unresolved Issues Regarding the Relationship between Peroxiredoxins and Genomic Stability

Here, we propose several different models whereby Tsa1 may safeguard the genome against instability. At present, the mechanism through which Tsa1 suppresses a broad spectrum of mutations has not been clearly established, although it is possible that one of the mechanisms described above (or some combination thereof) contributes to Tsa1’s role in lowering mutation rates. Further work on the genetic and biochemical levels is needed to clarify this complicated phenotype. In addition, it remains to be determined whether the loss of peroxiredoxins in other species enhances mutation rates, although it is clear that deletion of Prdx1 in mice enhances tumor incidence and can increase the likelihood of loss of heterozygosity in tumor suppressor genes in certain tissue types [77,96,97]. Once a mechanism for suppressing mutations is firmly established in baker’s yeast, critical next steps will be to clarify whether this mechanism is conserved across species, and to explore whether it represents a way through which peroxiredoxins decrease the incidence of malignancy in mammals. 

## Supplementary Materials

The following are available online at http://www.mdpi.com/2076-3921/7/12/177/s1, Experimental Methods.
