# Supplementary material for: Piecing Together How Peroxiredoxins Maintain Genomic Stability
Source: Antioxidants (Basel). 2018 Nov 28;7(12):177. doi: 10.3390/antiox7120177 (PMC6316004; doi:10.3390/antiox7120177)
Supplement: Supplementary file 1 [file antioxidants-07-00177-s001.pdf]

Supplementary Materials

Experimental Methods

Yeast Culture. Wild-type (BY4741) yeast or yeast lacking Tsa1 and Tsa2 (*tsa1 tsa2*) were culture as previously reported. Strains were transformed with p416-GPD (empty vector) or p416-GPD containing the respective genes of interest and maintained in SC-Ura medium.

Molecular Biology to Generate Tsa1 Active Site Variants and Clone Yeast Peroxidases. Most variants of Tsa1 and clones of yeast peroxidases are described in previous publications. Site-directed mutagenesis of Tsa1 to generate the C^{48}D variant was carried out in a manner similar to previously published methods using the Quick Change procedure and the following primers (substitutions are in lower case; F: 5′-GCCTTCACTTTCGTCgacCCAACCGAAATCATTGC; R: 5′-GCAATGATTTCGGTTGGgtcGACGAAAGTGAAGGC). Cloning of the FLAG-tagged Ctt1 and Ccp1 coding sequences into p416-GPD was conducted using standard molecular biology procedures using genomic DNA as a template. Primers used are shown in Supplementary Table 1. All clones were verified by restriction digestion and DNA sequencing prior to use.

Detection of FLAG-tagged Peroxidase Overexpression. Overexpressed peroxidases were detected using an immunoblot against the FLAG tag as previously described [1]. Blots were also probed for equal loading with an antibody against Pgk1.

Toxicity Assays with H_2O_2. Toxicity assays were carried out as previously described [2]. Briefly, stationary phase cultures of strains grown in SC-Ura were diluted to OD_{600} of 0.5 and serially diluted as indicated. Serial dilutions (4 μL) were plated on YPD medium or YPD medium containing 4 mM H_2O_2 and grown for 48 h at 30 °C.

Assessing Mutation Rates. To determine the effect of peroxidase overexpression on genomic stability in yeast, *tsa1 tsa2* strains expressing the tagged variants of Tsa1 were monitored for fluctuation in the *CAN1* gene as described elsewhere [3]. Briefly, yeast cultures were grown in SC-Ura medium overnight at 30°C to saturation. The following day, cultures were diluted by a factor of 10^6 in SC-Ura and grown for an additional 48 h at 30 °C. Subsequently, cultures (100 μL) were plated on SC-Ura-Arg plates containing 60 μg/mL canavanine sulfate and grown for 72 h at 30 °C. For viability measurements, samples were diluted by a factor of 10^5, from which 100 μL was plated on SC-Ura plates and grown for 48 h at 30 °C. Colonies on both fluctuation analysis plates and viability plates were counted. Mutation rates and corresponding 95% confidence intervals were determined by the median method.

Supplementary Table 1. Primers Used for Cloning FLAG-tagged Ctt1 and Ccp1.

Cct1-FLAG (SpeI/XhoI)

F: 5′-GGGG**ACTAGT***ATG*AACGTGTTCGGTAAAAAGAAG

R: 5′- GGGG**CTCGAG***TTA*TTTATCATCATCATCTTTGTAATCATTGGCACTTGCAATGGCACTTG

Ccp1-FLAG (SpeI/Xhol)

F: 5′-GGGG**ACTAGT***ATG*ACTACTGCTGTTAGGCTTTTAC

R: 5′-GGGG**CTCGAG***TTA*TTTATCATCATCATCTTTGTAATCTAAACCTTGTTCCTCTAAAGTCT

*Restriction sites are indicated in bold; the FLAG tag sequence in reverse primer is underlined; the start and the stop codons in forward and reverse primers, respectively, are italicized.

References

1 Naticchia, M.R.; Brown, H.A.; Garcia, F.J.; Lamade, A.M.; Justice, S.L.; Herrin, R.P.; Morano, K.A.; West, J.D. Bifunctional electrophiles cross-link thioredoxins with redox relay partners in cells. *Chem. Res. Toxicol.* **2013,** *26* (3), 490–497.

2. Allan, K.M.; Loberg, M.A.; Chepngeno, J.; Hurtig, J.E.; Tripathi, S.; Kang, M.G.; Allotey, J.K.; Widdershins, A.H.; Pilat, J.M.; Sizek, H.J.; Murphy, W.J.; Naticchia, M.R.; David, J.B.; Morano, K.A.; West, J.D. Trapping redox partnerships in oxidant-sensitive proteins with a small, thiol-reactive cross-linker. *Free Radic. Biol. Med.* **2016,** *101*, 356–366.

3. Huang, M.E.; Rio, A.G.; Nicolas, A.; Kolodner, R.D. A genomewide screen in Saccharomyces cerevisiae for genes that suppress the accumulation of mutations. *Proc. Natl. Acad. Sci. USA* **2003,** *100* (20), 11529–11534.
